# ICOS Co-Stimulation: Friend or Foe?

**DOI:** 10.3389/fimmu.2016.00304

**Published:** 2016-08-10

**Authors:** Daniel J. Wikenheiser, Jason S. Stumhofer

**Affiliations:** ^1^Department of Microbiology and Immunology, University of Arkansas for Medical Sciences, Little Rock, AR, USA

**Keywords:** T cell activation, co-stimulatory molecules, antibody production, parasitic and bacterial infection

## Abstract

Over the last 15 years, the inducible T cell co-stimulator (ICOS) has been implicated in various immune outcomes, including the induction and regulation of Th1, Th2, and Th17 immunity. In addition to its role in directing effector T cell differentiation, ICOS has also been consistently linked with the induction of thymus-dependent (TD) antibody (Ab) responses and the germinal center (GC) reaction. ICOS co-stimulation, therefore, appears to play a complex role in dictating the course of adaptive immunity. In this article, we summarize the initial characterization of ICOS and its relationship with the related co-stimulatory molecule CD28. We then address the contribution of ICOS in directing an effector T cell response, and ultimately disease outcome, against various bacterial, viral, and parasitic infections. Next, we assess ICOS in the context of TD Ab responses, connecting ICOS signaling to follicular helper T cell differentiation and its role in the GC reaction. Finally, we address the link between ICOS and human autoimmune disorders and evaluate potential therapies aiming to mitigate disease progression by modulating ICOS signaling.

## Introduction

During conventional T cell activation, interaction of the T cell receptor (TCR) with MHC class I or class II-peptide complexes initiates the cascade of T cell activation. However, an important secondary co-stimulatory signal must be delivered in concert with TCR stimulation in order to facilitate proper T cell activation (1). Previously, CD28 ligation had been shown to play a critical role in providing the “second signal” necessary to promote cellular proliferation and survival following T cell activation (2, 3), while CTLA-4 ligation served to mitigate this process (4), as recently reviewed elsewhere (5–7). The identification of the inducible T cell co-stimulator (ICOS) (8, 9) as a new member of the immunoglobulin (Ig) family of co-receptor molecules (8) led to a flurry of research regarding its role in adaptive immune responses.

First identified in humans (8) and shortly thereafter in mice (9), ICOS has significant homology to the co-stimulatory molecule CD28 and the immune-attenuator CTLA-4 (8, 10). Appropriately termed the inducible co-stimulator, ICOS is not constitutively expressed on resting T cells, but is instead rapidly induced following TCR cross-linking and/or CD28 co-stimulation (8, 11, 12). Along with CD28 and CTLA-4, ICOS is expressed on activated CD4 and CD8 T cells (13), suggesting that ICOS – analogous to CD28 and CTLA-4 activity – also regulates the adaptive T cell response. Thus, due to the large degree of homology between ICOS and other Ig family co-stimulatory molecules, in addition to T cell expression of ICOS, early research questioned whether ICOS played a homologous role to CD28 in the process of T cell activation and the initiation of cell-mediated adaptive immunity.

While a number of early studies evaluated the function of ICOS in T cell activation, proliferation, and differentiation, the predominant phenotype that emerged from the initial characterization of ICOS and ICOS ligand (ICOSL)-deficient mice revealed that this co-stimulatory molecule played a significant role in the generation of class-switched antibodies (Abs) against thymus-dependent (TD) antigens, which was attributed to a reduction in the number and size of germinal centers (GCs) in the spleen in the absence of ICOS signaling. Long-lived plasma cells (LLPCs) and memory B cells (MBCs) that have undergone class-switching and somatic hypermutation to increase Ab affinity are products of GC reactions; as these cell types and the Abs they produce are thought to be absolutely critical for maintaining life-long protection against pathogens following exposure or vaccination, or for contributing to the development of a number of autoimmune diseases, the most recent developments regarding the function of ICOS have focused on how ICOS–ICOSL interactions contribute to GC-derived Ab production.

Herein, we assess the relationship between CD28 and ICOS in regard to intracellular signaling events, and evaluate how these co-stimulatory molecules serve different roles in CD4^+^ T cell activation and proliferation. We then evaluate the influence of ICOS on the development of Th1 and Th2 type immunity in response to various bacterial, viral, and parasitic infections. Next, we discuss the early findings in *Icos*^−/−^ and *Icosl*^−/−^ mice that implicated ICOS in promoting TD antibody responses. We will then discuss more recent findings that suggest a role for ICOS signaling in the differentiation and maintenance of follicular helper T (Tfh) cells. To connect the function of ICOS during Tfh cell development with the humoral response as a whole, we will also assess how ICOS–ICOSL interactions modulate the GC reaction. Finally, a survey of potential therapeutic interventions regarding attenuation of ICOS signaling, and potential human considerations, is evaluated.

## ICOS and CD28

Although similar in structure, ICOS and CD28 appear to play non-redundant roles in modulating the activation of T cells (14, 15). Accordingly, in contrast to CD80 and CD86, which interact with CD28 and CTLA-4 (4), ICOSL binds ICOS exclusively (12, 16, 17). Interestingly, ICOS lacks the specific MYPPPY motif present in CD28 and CTLA-4 that has been shown to be necessary for interaction with CD80 and CD86 (8, 18). These data suggest the single receptor–ligand pair ICOS:ICOSL is likely regulated in a fashion distinct from CTLA-4 and CD28, as ICOSL expression itself has been shown to be downregulated following interaction with ICOS (19). ICOSL expression is largely restricted to professional antigen-presenting cells (APCs), including B cells [in which ICOSL is regulated by BAFFR and non-canonical NFκB signaling (20)], macrophages, and dendritic cells (DCs) (12, 17, 21, 22), but is also expressed by certain endothelial cells (23) and lung epithelium (24).

**Figure 1:**
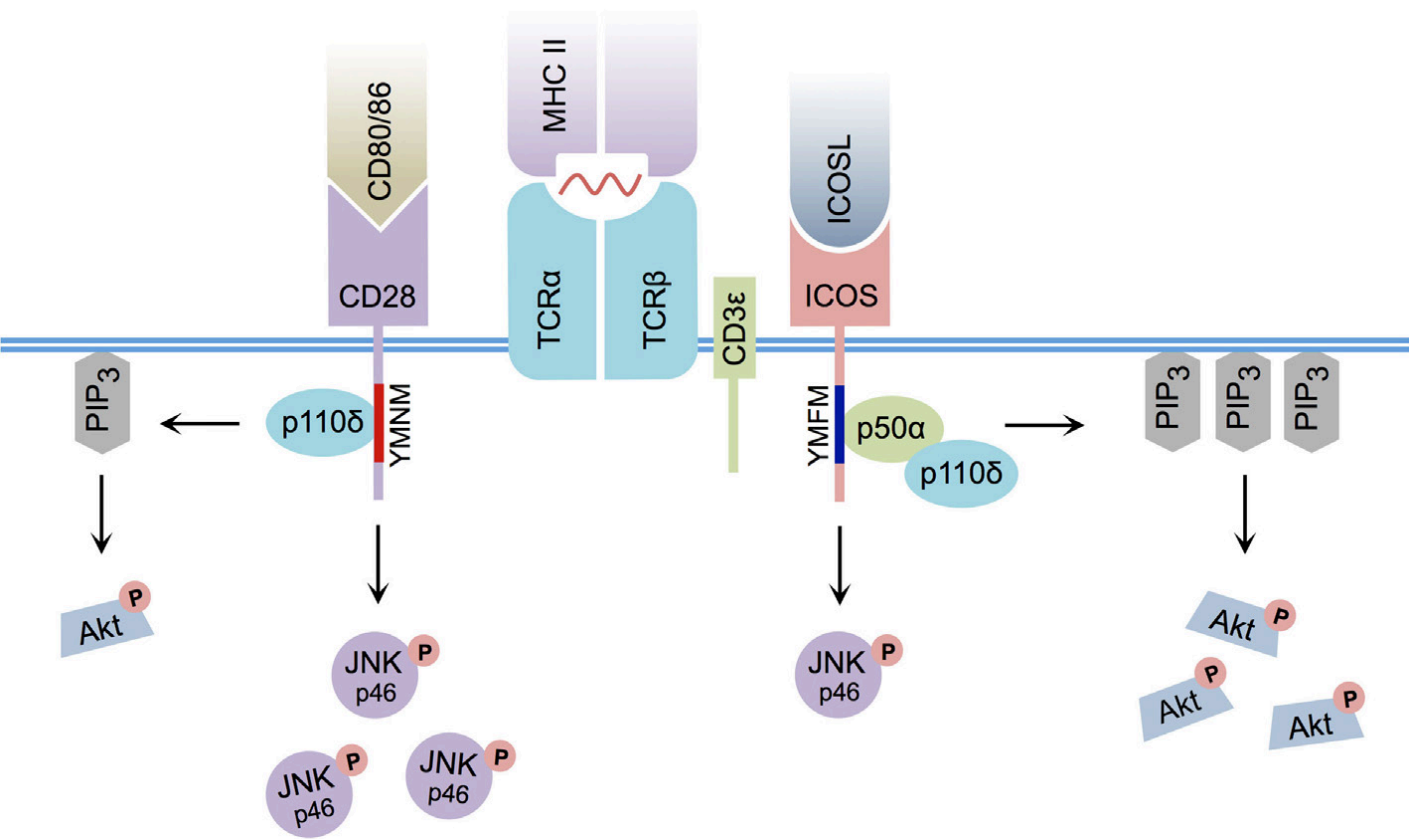
**Differential CD28 and ICOS signal transduction**. ICOS:ICOSL interaction induces PI3K p50α regulatory subunit recruitment via its YMFM signaling motif, resulting in strong PIP_3_ production and enhanced Akt phosphorylation. CD80/CD86:CD28 interaction induces less PIP_3_ production and weaker Akt phosphorylation. ICOS:ICOSL interaction induces less c-Jun N-terminal kinase (JNK) p46 phosphorylation relative to CD80/86:CD28 ligation.

Early research indicated that although ICOS and CD28 downstream signaling events were related, they were not identical (25) (Figure 1). For example, both ICOS and CD28 ligation induce the recruitment of class IA phosphatidylinositol 3-kinase (PI3K) (26, 27), a signaling molecule that leads to the production of membrane-bound phosphatidylinositol 3,4,5-trisphosphate (PIP_3_), culminating in the activation of Akt – a kinase known to promote cellular proliferation and survival (28). Similar to CD28 cross-linking, ICOS ligation can yield the recruitment of p50α (27) and p85α (29) regulatory subunits of PI3K, in conjunction with recruitment of the p110δ catalytic subunit (27, 29). However, ICOS ligation of activated CD4^+^ T cells was demonstrated to enhance production of PIP_3_ (27, 30) and induce stronger Akt phosphorylation than CD28 cross-linking (14, 27). Interestingly, the particular YxxM signaling motif YMFM found in the cytoplasmic tail of ICOS (YMNM in the case of CD28) (31) was responsible for preferential recruitment of the p50α subunit of PI3K (27), a regulatory subunit with inherently greater lipid kinase activity relative to p85α (32). Although ICOS signaling serves to specifically promote p50α recruitment and subsequent AKT activity, ICOS-mediated p85α recruitment has recently been shown to play a critical CD28-independent role in directing T helper cell effector fate by promoting the Tfh cell phenotype (29).

Although slight differences in PI3K signaling can explain some of the differences between CD28 and ICOS signaling, it cannot fully account for the specific activity of ICOS signaling itself, as disruption of the ICOS–PI3K interaction or deletion of PI3K components from T cells does not result in a complete phenocopy of *Icos*^−/−^ mice (15, 33, 34). Interestingly, differences in the pattern of MAPK activation between CD28 and ICOS have been reported, and ICOS co-stimulation has been shown to induce weaker phosphorylation of p46 c-Jun N-terminal kinase than CD28 (14). Furthermore, an additional intracellular signaling motif was recently identified in the cytoplasmic tail of ICOS that is not present in CD28. In response to simultaneous ICOS and CD3ε ligation, this cell-membrane proximal motif, termed IProx, was shown to recruit TBK1 (35), a member of the inhibitor of NF-κB kinase (IKK) family (36). While recruitment of TBK1 to the related co-stimulatory molecules CD28 and CTLA-4 was not observed following ligation with their respective ligands, ICOS:ICOSL-mediated recruitment of TBK1 was found to play an essential role in promoting the transition from an early Tfh cell to a GC-resident Tfh cell (35). As a whole, these data suggest that, although a commonality exists between CD28 and ICOS-mediated signal transduction, differences in downstream PI3K and MAPK signaling, as well as ICOS-specific TBK1 recruitment, likely contribute to their functional diversity.

## ICOS and T Cell Proliferation

Due to the shared homology between the co-stimulatory molecules CD28 and ICOS, early research sought to characterize the role of ICOS in T cell activation and proliferation. Interestingly, ICOS-deficient T cells exhibited a proliferation defect *in vitro* when compared with wild-type CD4^+^ T cells (37, 38). Furthermore, when immunized with keyhole limpet hemocyanin (KLH) adsorbed to alum, lymph nodes from *Icos*^−/−^ mice were found to be smaller in size when compared with wild-type mice, suggesting a similar proliferation defect *in vivo* (37). Similarly, during *Trichuris muris* and *Toxoplasma gondii* infection, CD4^+^ T cells isolated from *Icos*^−/−^ mice exhibited defects in activation and proliferation, but not differentiation (39). Additionally, when TCR transgenic T cells specific for ovalbumin (OVA) were polarized under Th1 or Th2 conditions *in vitro* and then transferred into naive recipients, ICOS was found to be necessary for expansion of both subsets (40). However, when ICOS-deficient mice were immunized with KLH in complete Freund’s adjuvant (CFA), no defect in cellular activation or proliferation was observed (37). These conflicting results led researchers to assess IL-2 production, an important step in promoting T cell clonal expansion (41). In contrast to CD28 ligation, multiple research groups discovered ICOS cross-linking did not induce IL-2 expression (8, 11, 14, 25, 26, 37), and instead induced the production of the anti-inflammatory cytokine IL-10 *in vitro* (8, 42). Thus, the role of ICOS in promoting CD4^+^ T cell proliferation is likely independent of IL-2 signaling, and the molecular basis for the role of this co-stimulatory molecule in promoting T cell expansion remains unclear. It is quite possible that ICOS signaling delivers a unique pro-survival or expansion signal not provided by CD28, but this remains to be determined. Furthermore, as differences in CD4^+^ T cell expansion have not been reported in every immunization or infectious disease model, the nature of the adjuvant or pathogen, as well as the degree of inflammation induced, may dictate the necessity of ICOS in T cell activation and clonal expansion – a topic we will touch upon further in the next section.

## ICOS and Infection

To better characterize the *in vivo* role of ICOS in the process of T cell differentiation during conditions relevant to human disease, a multitude of murine infection models, as well as strategies designed to disrupt ICOS signaling, have been investigated. As a whole, ICOS has been shown to regulate various T helper cell subsets during different infection scenarios, largely by promoting or inhibiting Th1 and Th2 immune responses (Table 1).

**Table 1:** **Summary of Th impact and disease outcome in various infection models when ICOS signaling is disrupted**.

Mouse strain	Genotype/treatment	Infection model	Th impact	Disease outcome
C57BL/6	*Icos*^−/−^	*M. tuberculosis*	Enhanced Th1	Accelerated control of splenic bacterial burden (43)
C57BL/6	*Icosl*^−/−^	*C. muridarum*	Enhanced Th1	Increased bacterial lung burden; increased lung pathology and weight loss (44)
C57BL/6	*Icos*^−/−^	*C. trachomatis*	No impact Th1 (primary) Enhanced Th1 (challenge)	Enhanced protection to secondary challenge; increased uterine inflammation (46)
C57BL/6	α-ICOS Ab	*S. mansoni*	Enhanced Th1	Increased egg granuloma size; greater hepatic immunopathology (47)
C57BL/6	*Icos*^−/−^	*S. mansoni*	Decreased Treg	No impact on parasite burden or egg granuloma formation (59)
C57BL/6	*Icos*^−/−^	*P. c. chabaudi*	Enhanced Th1 No Impact Treg	Accelerated control of acute parasitemia (48)
C57BL/6	*Icos*^−/−^	*S. enterica* (Typhimurium)	Decreased Th1	Increased liver and splenic bacterial burden; unable to resolve infection (49)
BALB/c	ICOS–Ig	*L. monocytogenes*	Decreased Th1	Increased splenic bacterial burden; lethality (50)
BALB/c	*Icos*^−/−^	*T. gondii*	Decreased Th1	Decreased brain inflammation (39)
C57BL/6	α-ICOSL Ab	*T. gondii*	Decreased Th1	Increased parasite burden (51)
C57BL/6	ICOS–Ig	LCMV	Decreased Th1	Not determined (52)
C57BL/6	*Icos*^−/−^	*H. polygyrus*	Decreased Treg	No impact on parasite burden or egg production (59)
129S4/SvJae	*Icos*^−/−^	*L. mexicana*	Decreased Th1 and Th2	Decreased immunopathology (53)
BALB/c	α-ICOS Ab	*T. spiralis*	Enhanced Th1 Decreased Th2	Lower muscle larvae burden; reduced intestinal immunopathology (67)
BALB/c	α-ICOSL Ab	*L. major*	Decreased Th2	Delayed footpad swelling (68)
BALB/c	*Icos*^−/−^	*N. brasiliensis*	Decreased Th2	Enhanced parasite egg production (68)
BALB/c	*Icos*^−/−^	*T. muris*	Decreased Th2	Delayed worm expulsion (39)
C57BL/6	*Icos*^−/−^	*B. malayi*	No impact Th2 (chronic inf.) Decreased Th2 (immunized)	Not determined (69)

## Th1 Immunity

In the context of *Mycobacterium tuberculosis* infection, for example, mice lacking expression of ICOS exhibited evidence of enhanced Th1 immunity, producing a significantly greater number of CD4^+^IFN-γ^+^ T cells in the spleen and lungs during later stages of infection. Concomitantly, regulatory T cell (Treg) frequency was significantly reduced in ICOS-deficient mice in this infection model. In the end, ICOS deficiency led to enhanced control of *M. tuberculosis* infection in the spleen, but not the lungs (43).

ICOS ligand-deficient mice infected with *Chlamydia muridarum* also produced a significantly stronger Th1 response, with enhanced production of IFN-γ, IL-6, and TNF-α. Furthermore, the authors observed lower production of the anti-inflammatory cytokines IL-10 and TGF-β in ICOSL-deficient mice. Similar to *M. tuberculosis* infection, *Icosl*^−/−^ mice infected with *C. muridarum* exhibited higher bacterial lung burden and showed greater evidence of lung pathology, as well as losing more body weight than wild-type control mice (44). Additional research has also linked ICOS-mediated PI3K signaling with the induction of Th17 responses during *C. muridarum* infection. In this study, transgenic mice harboring an ICOS signaling mutation (preventing PI3K from interacting with ICOS) mounted a defective Th17 response compared with wild-type mice, culminating in decreased control of bacterial burden in the lungs (45).

On the other hand, during *Chlamydia trachomatis* genital tract infection, *Icos*^−/−^ mice controlled primary infection similarly to wild-type mice. However, following resolution of acute infection, ICOS-deficient mice exhibited increased cellular infiltrate and inflammation within the uterine horns. Surprisingly, *Icos*^−/−^ mice showed enhanced protection to secondary *C. trachomatis* challenge, with a greater frequency of IFN-γ^+^ T cells observed in the uterus of *Icos*^−/−^ mice (46).

In the case of *Schistosoma mansoni* infection, wild-type mice treated with α-ICOS neutralizing Abs developed larger egg granulomas and displayed evidence of enhanced hepatic immunopathology. Increased production of IFN-γ concomitant with decreased IL-10 secretion in α-ICOS Ab-treated mice suggests an enhanced Th1 response likely mediated the associated hepatic pathology (47).

In agreement with evidence of enhanced Th1 immunity in *Icos*^−/−^ mice, during *Plasmodium chabaudi chabaudi* AS infection, ICOS served to dampen the Th1 response, as *Icos*^−/−^ mice produced more IFN-γ secreting CD4^+^ and CD8^+^ T cells and had higher serum IFN-γ than wild-type mice. Additionally, enhanced expansion of CD25^+^T-bet^+^ effector CD4^+^ T cells was observed in ICOS-deficient mice. The augmented Th1 response observed in *Icos*^−/−^ mice led to lower peak parasitemia and accelerated control of acute *P. c. chabaudi* AS infection relative to wild-type mice (48).

There are, however, examples in which ICOS appears to promote Th1 immunity, such as systemic *Salmonella enterica* (serovar Typhimurium) infection. In this model, ICOS-deficient mice were defective in CD4^+^ T cell IFN-γ production, despite having no defect in total CD4^+^ T cell activation. Accordingly, *Icos*^−/−^ mice had substantially increased bacterial burden in the liver and spleen at days 14 and 21 post-infection compared with wild-type mice. In the end, *Icos*^−/−^ mice were unable to fully resolve *S. enterica* infection, and continued to harbor bacteria in the spleen at day 36 post-infection (49).

In the same manner, disruption of ICOS signaling during oral *Listeria monocytogenes* infection via ICOS–Ig treatment led to decreased Th1 immunity and significantly increased splenic bacterial burden and lethality. Interestingly, in ICOS–Ig treated mice, CD4^+^ T cells were unable to produce IFN-γ in response to *ex vivo* stimulation with heat-killed *Listeria*, contrary to control-Ig treated mice. These data suggest the magnitude of the Th1 response was blunted when ICOS signaling was impaired (50).

In the context of a strong Th1-inducing parasite, such as *T. gondii*, ICOS and CD28 appear to play a redundant role in the induction of Th1 immunity. In the absence of CD28, mice were still capable of producing IFN-γ. However, disruption of ICOS signaling via α-ICOSL Ab treatment significantly reduced production of IFN-γ while simultaneously increasing parasite burden (51). In *T. gondii*-infected *Icos*^−/−^ mice (with intact CD28 signaling), no defect in T cell-derived IFN-γ was observed. Instead, the absence of ICOS signaling decreased CD4^+^ T cell expansion during acute and chronic infection leading to reduced inflammation, but did not impact parasite burden, suggesting a redundant role concerning IFN-γ production but a unique role for ICOS in promoting T cell expansion (39). In contrast, disruption of ICOS signaling via ICOS–Ig fusion protein during LCMV infection diminished IFN-γ production, and this phenotype was potentiated in ICOS–Ig-treated *Cd28*^−/−^ mice (52). These data suggest that, in response to LCMV infection, ICOS and CD28 may synergistically promote Th1 immunity.

Additionally, ICOS may simultaneously promote ongoing Th1 and Th2 immune responses. During cutaneous *Leishmania mexicana* infection, for example, researchers observed a slight reduction in IFN-γ and IL-4 production in *Icos*^−/−^ mice at week 6 post-infection, while the production of both cytokines was substantially reduced in ICOS-deficient mice by week 12 post-infection. This reduced inflammation did not lead to an increase in parasite burden; in fact, lesions were initially slower to develop in *Icos*^−/−^ mice, but by week 12 post-infection, lesions were similar to those observed in wild-type mice. Despite normal lesion formation, however, tissue damage was less severe in *Icos*^−/−^ mice at week 12 post-infection compared with wild-type mice (53), suggesting that, in the case of chronic *L. mexicana* infection, ICOS signaling served to enhance immune-mediated pathology.

Although ICOS is seemingly capable of alternatively promoting or repressing Th1 responses under different infection conditions, these divergent phenotypes may in part be explained by ICOS-dependent Treg induction (54, 55). For instance, it is well established that CD4^+^ T cells from naive mice that express high levels of ICOS also produce the greatest amount of IL-10 (56), a cytokine often produced by Tregs. In a different study, administration of a toll-like receptor (TLR) 4 ligand enhanced the frequency of IL-10-producing ICOS^+^CD4^+^Foxp3^+^ Tregs in lung draining lymph nodes following birch pollen allergen nasal challenge, ameliorating the development of severe disease (57). Similarly, *Icos*^−/−^ mice tolerized with intranasal OVA developed significantly fewer splenic and lung-resident CD4^+^Foxp3^+^ Tregs at day 6 post-challenge relative to wild-type mice (58). Together, these data connect ICOS expression with Treg functionality in models of allergic challenge, as well as during naive conditions. Additionally, ICOS has been directly linked with Treg induction during infection. *Icos*^−/−^ mice infected with *Heligmosomoides polygyrus* or *S. mansoni*, for instance, exhibited delayed expansion and significantly fewer total CD4^+^Foxp3^+^ Tregs in the mediastinal lymph node and spleen, respectively, when compared with wild-type mice (59). Thus, the ability of ICOS to influence Treg induction further complicates its role in regulating CD4^+^ T cell differentiation, and suggests ICOS signaling may be mutually important in preventing immune-mediated pathology, as well as inducing pro-inflammatory CD4^+^ T cells. However, the regulatory function of ICOS is not limited to Treg cell induction and IL-10 production, as *Icos*^−/−^ mice display an enhanced Th1 response after infection with *P. c. chabaudi* AS, despite similar numbers of splenic Tregs and IL-10 secreting CD4^+^ T cells as wild-type mice (48).

Altogether, these data strongly implicate ICOS in modulating Th1 immune responses. It is, however, difficult to isolate the precise contribution of ICOS in promoting or regulating Th1 immunity, as ICOS signaling likely influences T cell proliferation, as well as effector cell fate decisions, including Treg induction, simultaneously. As there is a degree of redundancy between CD28 and ICOS signaling, perhaps the integration of ICOS and CD28 co-stimulatory signals, as well as signals from other surface proteins, such as the TCR, cytokine receptors, and other co-stimulatory molecules, dictate the significance of ICOS:ICOSL signals in promoting or regulating T helper cell differentiation. As various pathogens are capable of influencing APC activation status by modulating CD80 (60), CD86, and ICOSL (61–64) surface expression, it is not unreasonable to suggest the relative ratio of ICOS:CD28 co-stimulation a CD4^+^ T cell receives varies greatly based on the infectious agent. The relevance of ICOS signaling in CD4^+^ helper T cell differentiation, therefore, may also be connected to the innate signaling events responsible for promoting APC maturation and subsequent upregulation of CD28 co-stimulatory ligands and ICOSL.

## Th2 Immunity

One of the original hypotheses for the function of ICOS suggested that it played an important role in driving Th2 immunity, as ICOS expression is preferentially sustained on Th2 immune cell subsets (11, 26, 39), while *in vitro* disruption of ICOS signaling yields decreased IL-4 production (37–39, 65, 66). Likewise, in the context of *Trichinella spiralis* (67) and *Leishmania major* (68) infection, treatment of wild-type mice with anti-ICOS or anti-ICOSL Abs served to diminish the Th2 immune response, reducing the production of IL-4, IL-5, and IgE. However, during *T. spiralis* infection, anti-ICOS Ab treatment also simultaneously promoted TNF-α, IFN-γ, and IL-10 production. While disruption of ICOS signaling did not impact worm load in the intestine, it did substantially reduce immunopathology within the intestine itself. Interestingly, anti-ICOS Ab-treated mice showed significantly lower larvae burden in striated muscle, a site of secondary colonization, when compared with control mice (67).

Blunted Th2 immunity was also observed in *Nippostrongylus brasiliensis*-infected *Icos*^−/−^ mice, in which parasite egg production was enhanced in the absence of ICOS (68). Similarly, disruption of ICOS signaling in *Cd28*^−/−^ mice via ICOS–Ig treatment diminished IL-4 and IL-5, as well as IFN-γ and IL-10 production in *N. brasiliensis*-infected mice, but surprisingly did not hinder or delay worm expulsion (52).

Although ICOS appears to play an important role in promoting Th2 immunity, in the case of chronic *Brugia malayi* infection, *Icos*^−/−^ mice were not defective in their ability to produce IL-4 or IgE compared with wild-type mice. Furthermore, eosinophil recruitment to the peritoneal cavity was not defective in the absence of ICOS. However, during primary immunization with *B. malayi* antigen, fewer IL-4 producing Th2 cells were identified in *Icos*^−/−^ mice at day 10 post-immunization (69), suggesting that ICOS may be more important during the priming phase of Th2 immune responses.

In a similar fashion, although defective Th2 immunity was observed during acute *Trichuris muris* infection, *Icos*^−/−^ mice produced more IL-13 and IL-5 at day 35 post-infection compared with wild-type mice, suggesting that Th2 immunity was slower to develop in the absence of ICOS. Worm burden data corroborate this idea, as *Icos*^−/−^ mice continued to harbor worms at day 18 post-infection – a time-point at which wild-type mice had eliminated the parasite. However, by day 54 post-infection, *Icos*^−/−^ mice had largely controlled worm burden, suggesting that the onset of Th2 immunity was delayed, but not absent, in *Icos*^−/−^ mice (39). Similar to the studies with *T. gondii*-infected *Icos*^−/−^ mice, the delayed Th2 response was attributed to reduced expansion of Th2 polarized CD4^+^ T cells.

Collectively, the development of Th2 immunity appears to be more critically reliant on ICOS signaling than Th1 immune responses. In all infection models reviewed herein, disruption of ICOS signaling led to poor CD4^+^ T cell Th2 polarization and diminished IL-4 production, which may or may not have been due to reduced expansion of CD4^+^ T cells in all cases. Similar to the results seen with infection models that induce a Th1 response, the absence of ICOS signaling did not completely suppress the development of Th2 immunity. In a number of circumstances, anti-worm Th2 immunity in *Icos*^−/−^ mice was acutely defective, but ultimately played an important, albeit delayed, role in controlling worm burden. In the case of the worm infections described above, the late enhancement of the Th2 response coincided with the development of the larvae into adult worms, indicating changes in antigen or persistence of antigen may allow for the late expansion of the Th2 response in these models. Similarly, variation in antigen or antigen load may also account for the variable requirement of ICOS signaling in Th1 cell induction in previously discussed Th1 infection models. Nevertheless, the studies presented here indicate that ICOS co-stimulation plays an important role in either enhancing or regulating Th cell expansion and/or differentiation after infection.

## ICOS Promotes TD Antibody Responses

Although ICOS can modulate Th1/Th2 differentiation during infection, early observations connected ICOS co-stimulation with Ab production. During the initial phenotypic characterization of ICOS, for instance, researchers noted that, within secondary lymphoid tissue, ICOS expression was largely restricted to GC light zones (8), an area within B cell follicles known to be populated by CD4^+^ T cells (70). To address the contribution of ICOS to Ab production, *Icos*^−/−^ or *Icosl*^−/−^ mice were immunized with a number of thymus-independent (TI) and TD antigens.

When *Icosl*^−/−^ mice were immunized with the TI type I (TI-I) antigen nitrophenol (NP)-conjugated LPS no defect in the production of NP-specific IgM or IgG3 Abs was noted (66). Although a number of TLR agonists, including LPS, are capable of upregulating ICOSL expression on splenic B cells (64), the absence of ICOSL expression did not affect B cell activation in response to NP-LPS immunization. Likewise, immunization of *Icosl*^−/−^ mice with the TI type II (TI-II) antigen NP-Ficoll yielded no defect in NP-specific IgM or IgG3 Abs relative to wild-type control mice. However, while immunization of *Icosl*^−/−^ mice with the TD antigen NP_21_-conjugated chicken globulin (CG) adsorbed to alum led to no defect in NP-specific IgM Abs, a substantial decline in IgG1 and IgG2a production was observed (66). Similarly, following immunization with the TD antigen sheep red blood cells (SRBC), treatment of mice with α-ICOSL Abs did not impact production of SRBC-specific IgM, but significantly diminished IgG1, IgG2a, and IgG2b-specific Abs (71). Ultimately, SRBC-boosted *Icos*^−/−^ mice (72) and NP_21_-CGG boosted *Icosl*^−/−^ mice (66) failed to form secondary GCs when compared with wild-type mice.

In the case of the TI anti-polysaccharide response to whole *Streptococcus pneumoniae* immunization, no defect in IgG Ab production was observed in *Icos*^−/−^ mice (73). Perhaps not surprisingly, given that TI antigens often induce abortive GCs and instead promote isotype-switched Ab production through an extrafollicular response (74, 75), this class-switched Ab was determined to be largely extrafollicular in nature. However, immunization of *Icos*^−/−^ mice with *S. pneumoniae* cell-wall polysaccharide conjugated to PspA (an *S. pneumoniae* surface protein) yielded defective anti-polysaccharide IgG production relative to wild-type mice, indicating that direct conjugation of a TI antigen to protein results in a dependency on a GC reaction and ICOS signaling to generate isotype-switched Abs (73). Collectively, although ICOS did not appear to play an essential role in TI Ab production or IgM-specific Ab formation against protein or a haptenated protein antigen, production of TD class-switched Abs appeared to require intact ICOS signaling. Furthermore, ICOS performed an important function in mediating the secondary humoral response to previously encountered TD antigens.

To further interrogate the role of ICOS in TD humoral immunity, the Ab response to various model Ag-adjuvant combinations was assessed in ICOS-deficient mice. In the presence of either alum or CFA, for example, *Icos*^−/−^ and *Icosl*^−/−^ mice exhibited a profound defect in the IgG1-specific Ab response to KLH when compared with wild-type mice (37, 38, 65), with a similar reduction in GC formation (38, 65). In agreement with these data, immunization of *Icos*^−/−^ mice with 2,4,6-trinitrophenol (TNP)-conjugated KLH in incomplete Freund’s adjuvant (lacking *Mycobacterium* cell-wall components) or alum yielded gross defects in the production of IgG2a and IgG1-specific TNP Abs, but did not diminish TNP-specific IgM production. Accordingly, GCs were smaller in size and number in *Icos*^−/−^ mice compared with wild-type mice. However, when immunized with TNP–KLH in CFA, ICOS-deficient mice were not defective in the production of TNP-specific IgG isotype Abs, and subsequent GC size was not impaired, although a defect in the total number of GCs was observed in *Icos*^−/−^ mice relative to wild-type mice (76).

Collectively, these data provide seemingly conflicting results concerning the necessity of ICOS in TD Ab responses. While the use of a strong inflammatory adjuvant, such as CFA, which contains components that can serve as TLR agonists, could explain why TNP-KLH can elicit a class-switched Ab response in the absence of ICOS, it does not explain why immunization with un-conjugated KLH in CFA cannot. Interestingly, only the IgG1, but not IgG2a, response against KLH was significantly reduced in *Icos*^−/−^ and *Icosl*^−/−^ mice after immunization with KLH in CFA (38, 65). These data suggest that, similar to the result of TNP-KLH in CFA immunization, the inclusion of CFA can result in the production of class-switched Abs of certain isotypes in the absence of ICOS signaling. As the Th2-associated cytokine IL-4 is required for isotype switching to IgG1, and ICOS signaling has been implicated in Th2 differentiation (37–39, 65, 66), the failure to induce a strong IgG1 response against KLH in CFA may be partially due to impaired Th2 development. In support of this idea, *Icos*^−/−^ and *Icosl*^−/−^ mice have been shown to have lower serum baseline concentrations of IgG1 (38, 65). Also, differences in the Ab response against KLH and TNP-KLH in CFA could be explained by the nature of the antigen itself (protein alone versus hapten–protein complex, respectively). As only the antibody response against the hapten was measured in the TNP-KLH in CFA immunization studies, the KLH-specific Ab response could be defective in the *Icos*^−/−^ mice as well. Also, as IgG production was only assessed in the short-term for these particular studies (between days 7 and 14 post-immunization), it is possible extrafollicular isotype-switched Ab production could account for the TNP-specific IgG1 produced in *Icos*^−/−^ mice immunized with TNP-KLH in CFA. In future studies, long-term maintenance of isotype-switched Ab titers, perhaps, may prove a more useful metric for assessing the role of ICOS in TD Ab production.

In connection to its role in promoting TD Ab responses, ICOS may also regulate Ab affinity maturation – an important function of the GC reaction. In addition to producing significantly lower amounts of NP-specific IgG1 Abs than wild-type mice following NP-conjugated OVA immunization, the NP_4_/NP_23_ IgG1 ratio was significantly lower in *Icosl*^−/−^ mice at 6 weeks post-immunization, indicating a defect in Ab affinity maturation occurred in the absence of ICOS signaling. However, the NP_4_/NP_23_ IgG1 ratio did not differ significantly at 10 weeks post-immunization relative to wild-type mice, suggesting that between weeks 6 and 10 post-NP-OVA immunization affinity maturation proceeded in an ICOS-independent manner (65). As the absence of ICOS signaling does not completely prevent GC formation but rather reduces the size and number of GCs (77), perhaps the few GCs present in *Icosl*^−/−^ mice are capable of slowly producing affinity-matured NP-specific IgG1, such that by 10 weeks post-immunization the NP_4_/NP_23_ IgG1 ratio is similar between wild-type and *Icosl*^−/−^ mice. In contrast to this result, *P. c. chabaudi* AS-infected *Icos*^−/−^ mice exhibited a profound defect in parasite-specific IgG affinity maturation at weeks 3 and 9 post-infection, suggesting that affinity maturation during murine *Plasmodium* infection is critically dependent on continuous ICOS signaling (48).

While these data provide conflicting results concerning the role of ICOS in promoting TD Ab responses, it ultimately indicates that ICOS is a critical component in the production of isotype-switched Abs, particularly affinity-matured ones. Because the nature of the adjuvant or immunogen – hapten, protein, or whole pathogen – vary widely across experiments, the precise role of ICOS in promoting these Ab responses is not immediately clear. Nonetheless, the presence of ICOS^+^ CD4^+^ T cells within GCs suggests an important role for this molecule in supporting the GC reaction. Subsequent research, therefore, has explored the link between ICOS and CD4^+^ T cells in the larger context of TD Ab responses.

## ICOS and Tfh Cell Differentiation

Recently, ICOS has been directly implicated in the induction of a specific CD4^+^ T cell effector subset known as Tfh cells (15, 29, 71, 78–81). Within the GC, Tfh cells play a critical role in promoting the selection and survival of B cells expressing high-affinity B cell receptors, a process that ensures only B cells with the highest affinity for a given antigen differentiate into plasma cells and MBCs (70). The Tfh cell program of differentiation is critically reliant on Bcl6 expression (82, 83), a transcriptional co-repressor that antagonizes Blimp-1 – a transcription factor responsible for dictating non-Tfh CD4^+^ T cell effector programs. Successful repression of Blimp-1 by Bcl6 promotes the Tfh cell phenotype, and allows a pre-Tfh cell to upregulate additional molecules required for Tfh cell differentiation (84).

A critical component of the Tfh cell phenotype is expression of the chemokine homing receptor CXCR5, which binds CXCL13 produced by follicular DCs to promote the migration of Tfh cells to the B cell follicle. In addition to CXCR5, Tfh cells also express the co-inhibitory receptor PD-1, which serves to prevent excessive, unchecked proliferation. Tfh cells that have established residence within the GC light zone itself (GC Tfh cells) adopt a PD-1^high^CXCR5^high^ phenotype, and are phenotypically distinct from non-GC-resident Tfh cells. Additionally, Tfh cell production of the cytokine IL-21 is a key component in promoting the GC reaction, as well as reinforcing the Tfh cell phenotype itself in an autocrine manner (84).

Although Tfh cells have been published on extensively in the last several years, the link between ICOS and CXCR5^+^ CD4^+^ T cells had been the subject of earlier research. For example, it had been previously demonstrated that following immunization with SRBCs (71) or type II collagen (78), treatment of mice with α-ICOSL Abs abrogated expression of CXCR5^+^ on CD4^+^ T cells. Additionally, during secondary SRBC immunization, α-ICOSL Ab treatment similarly prevented induction of CXCR5^+^ on CD4^+^ T cells when compared with control mice, suggesting that ICOS supports T cell CXCR5 expression during primary and secondary GC responses (71).

While these studies highlighted the importance of ICOS signaling in promoting CXCR5 expression and possibly the generation of Tfh cells, the source and timing requirements of ICOSL co-stimulation were not fully understood. Continued efforts to better understand how ICOS influences the Tfh cell program have yielded novel insights into the process of Tfh cell differentiation. For instance, a recent report demonstrated CD8α^−^ DCs upregulate ICOSL expression following stimulation with polyI:C or LPS. This CD8α^−^ DC subset was subsequently shown to be important for induction of Tfh cells *in vitro* and *in vivo* (85). Previous research had also investigated the role of ICOS in the early stages of Tfh cell induction during DC:T cell interactions following lymphocytic choriomeningitis virus (LCMV) infection. Transfer of *Icos*^−/−^ LCMV-specific SMARTA CD4^+^ T cells into wild-type mice followed by LCMV infection resulted in a marked defect in Bcl6 and CXCR5 expression at days 3 and 4 post-infection relative to wild-type SMARTA T cells, suggesting that ICOS plays an essential role in the induction of Tfh cells. Furthermore, B cell-deficient μMT mice infected with LCMV were shown to have no defect in CD4^+^ T cell expression of Bcl6 or CXCR5 at day 3 post-infection, suggesting that B cells are not exclusively responsible for promoting early Tfh cell induction. However, by day 4 post-infection, Tfh cell frequency in μMT mice had declined significantly relative to wild-type mice, suggesting that interaction with B cells is necessary for maintenance of Tfh cells. Collectively, the authors concluded that ICOSL signaling from DCs is initially required for early Tfh cell priming, while cognate B cells provide a secondary source of ICOSL to reinforce the GC Tfh cell phenotype (79).

In agreement with a role for B cells in Tfh cell maintenance, B cell ICOSL expression was found to be essential for sustained Tfh cell:B cell engagement within the GC itself (86), suggesting that ICOS:ICOSL interactions increase the duration of T:B cell contact, consequently impacting affinity maturation, B cell survival, and plasma cell differentiation. In a different study, despite no defect in generating Bcl6^+^ pre-Tfh cells (early Tfh cells that have not yet entered the B cell follicle), when MHC-II expression was restricted to CD11c^high^ DCs, PD-1^++^ GC Tfh cells were unable to form following NP-OVA-alum immunization (87). Together, these data indicate that B cells deliver a critical ICOSL signal necessary to promote full GC Tfh cell maturation. Indeed, Tfh cells polarized *in vitro* in the presence of B cells produced substantially more IL-21, a cytokine linked to the GC reaction, than Tfh cells co-cultured with DCs alone (87). Furthermore, co-culture of wild-type or *Icos*^−/−^ α-CD3ε-stimulated memory CD4^+^ T cells with ICOSL-expressing B cells revealed enhanced IL-21 mRNA synthesis in the presence of ICOS signaling (88), indicating that B cells support the production of IL-21 via ICOS:ICOSL interactions. Interestingly, while transfer of T cells alone into immunodeficient SCID mice was not sufficient for proper Tfh cell development following SRBC immunization, co-transfer of T and B cells yielded Tfh cell differentiation; administration of α-ICOSL Abs disrupted Tfh cell differentiation under these conditions (71). As a whole, these data suggest that cooperative DC and B cell ICOSL co-stimulation serves a central role in the process of Tfh cell induction and maintenance, respectively. Whether ICOSL signaling from other cell types within the GC, such as DCs or follicular DCs, is also necessary to maintain or promote Tfh cell polarization during the GC reaction is still unknown.

Inducible T cell co-stimulator signaling, however, may not be critical for initial Tfh cell differentiation under all circumstances. For instance, *Icos*^−/−^ OT-II cells co-transferred with NP-specific B cells were not defective in Bcl6, c-Maf, or IL-21 expression following NP-OVA immunization. Nonetheless, *Icos*^−/−^ T cells were unable to fully downregulate CCR7 – an essential step in assuming the GC Tfh cell phenotype – and upregulate CXCR5, resulting in an inability of ICOS-deficient T cells to migrate into the B cell follicle. Furthermore, ICOS signaling was required to maintain the GC reaction, as α-ICOSL Ab treatment on day 6 post-NP-OVA immunization led to a loss of Tfh cells and the GC itself (80).

Indeed, in the context of murine *Plasmodium* infection, ICOS appears to play a more substantial role in promoting Ab production late in the infection, suggesting that B cells may be the most important source of ICOSL stimulation for Tfh cell maintenance. Prior to day 6 post-infection, for example, *Icos*^−/−^ CD4^+^ T cells were not defective in their ability to express the canonical Tfh cell markers Bcl6, PD-1, CXCR5, or IL-21. However, by week 3 post-infection, there was a substantial reduction in CD4^+^ T cells expressing these Tfh cell markers in *Icos*^−/−^ mice, and the remaining Tfh cells were unable to form PD-1^++^CXCR5^++^ GC Tfh cells. The end result was a drastic decrease in the total number of splenic GCs in ICOS-deficient mice relative to wild-type mice. Quite surprisingly, *Icos*^−/−^ mice were not defective in production of merozoite surface protein-1 42 kDa fragment (MSP-1_42_)-specific IgM or IgG isotype Abs at week 3 post-infection. However, the GC defect in *Icos*^−/−^ mice resulted in a significant decrease in both quantity and quality of parasite-specific Abs by week 6 post-infection (48). As a whole, the complexity of the Ab response to *Plasmodium* (89), coupled with the parasite’s ability to induce a strong extrafollicular Ab response, may help explain why ICOS is less critical in promoting early isotype-switched Ab production during *P. c. chabaudi* AS infection. Although initial Tfh cell induction was not impaired in *Icos*^−/−^ mice, proper GC Tfh cell differentiation and Ab affinity maturation required intact ICOS signaling (48), providing further evidence that GC Tfh cell formation necessitates B cell-specific ICOSL co-stimulation.

There are additional circumstances in which ICOS:ICOSL signaling is not required for differentiation of Tfh cells or the establishment of GCs. For instance, in response to NP-OVA immunization in alum, when antigen is abundant and antigen-specific B cells are present in high frequency, Tfh cell differentiation, GC formation, and production of class-switched, affinity-matured NP-specific IgG1 Abs is not impaired when cognate B cells are deficient in ICOSL expression (90). These data suggest ICOS co-stimulation by cognate B cells is only required under conditions when antigen is limiting. This idea is supported by additional findings indicating that DCs can support Tfh cell differentiation in the presence of excess antigen, even when B cell antigen presentation is abolished, indicating that a unique B cell-specific signal is not required for Tfh cell development under all conditions. However, this study further demonstrated that Tfh cells needed to interact with cognate B cells to form GCs, as DC-Tfh cell interactions could not promote GC formation on their own (91). Interestingly, not only the interaction of T cells with APCs but also the duration of these interactions has been shown to influence differentiation of Tfh cells, recruitment of Tfh cells into GCs, and subsequent function of Tfh cells within the GC (77). As such, ICOS:ICOSL interactions may help facilitate and extend the length of these interactions, particularly with cognate B cells, when antigen is limiting.

While ICOS:ICOSL signaling between cognate T and B cells is critical for progression to and maintenance of the GC Tfh cell phenotype, B cells may also play a more unconventional role in promoting Tfh cell differentiation – potentially in the form of non-cognate help. After noting that *Icos*^−/−^ OT-II CD4^+^ T cells expressed less CXCR5 and failed to migrate beyond the T:B cell border, researchers sought to determine if the lack of CXCR5 impacted follicular homing of *Icos*^−/−^ pre-Tfh cells. However, forced CXCR5 or Bcl6 expression by *Icos*^−/−^ OT-II cells did not enhance their ability to migrate beyond the T:B cell border into the B cell follicle. Strikingly, neither ICOSL expression by DCs or cognate B cells was found to facilitate proper follicular recruitment of early Tfh cells. Instead, ICOS:ICOSL interactions between T cells and non-cognate B cells facilitated migration of T cells into the GC. In the end, non-cognate B cells at the T:B cell border were found to provide an important ICOSL signal that led to T cell-intrinsic PI3K signaling, ultimately promoting T cell motility (92).

Relative to model antigen:adjuvant-based immunization strategies, the presence of an actively replicating pathogen dynamically alters the distribution and abundance of antigen available to the immune system. As such, infectious conditions add additional layers of complexity to the interpretation of data. While under certain circumstances, ICOS plays a critical role in priming Tfh cell induction, different inflammatory conditions and/or model systems appear to preclude the necessity of ICOS in promoting Tfh cell formation. However, as a whole, ICOS signaling critically supports GC Tfh cell functionality. Altogether, although ICOS clearly regulates the processes of Tfh cell differentiation, maintenance, and function, the particular timing of requisite ICOSL signal delivery appears to vary widely based on the model system employed.

## ICOS Signaling and Tfh Cells

**Figure 2:**
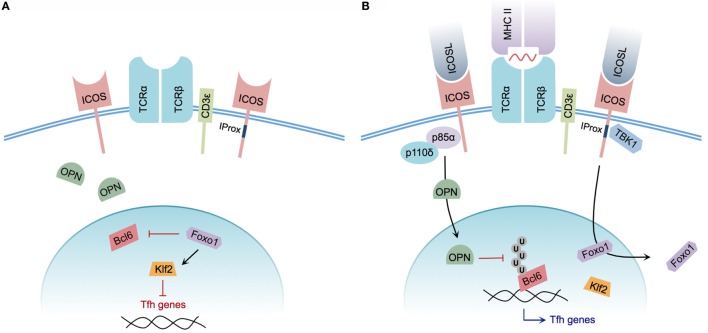
**Model of ICOS-mediated Tfh cell differentiation**. **(A)** CD4^+^ T cell without ICOS ligation. Osteopontin (OPN) is sequestered in the cytoplasm; Foxo1 represses Bcl6 while promoting Klf2-mediated repression of Tfh cell-associated genes. **(B)** CD4^+^ T cell with engaged TCR and ICOS co-stimulation. The p85α regulatory subunit of PI3K promotes OPN translocation to the nucleus, preventing ubiquitination and promoting the stability of Bcl6. Foxo1 is exported from the nucleus, relieving Klf2-mediated repression of Tfh cell genes. Integrated CD3ε and ICOS ligation induces recruitment of TBK1 to the intracellular IProx domain of ICOS, facilitating Tfh to GC Tfh transition.

Mechanistically, the signals underlying Tfh cell differentiation are not yet fully understood. However, the specific contribution of ICOS to the process of Tfh cell induction has recently come under greater scrutiny (Figure 2). The significance of ICOS-mediated PI3K signaling, for example, had been well characterized and described previously (14, 27). A specific role for PI3K recruitment in the context of Tfh cell development was demonstrated by generating knock-in mice expressing a mutant ICOS receptor harboring a cytoplasmic tail incapable of recruiting PI3K while retaining the ability to promote Ca^2+^ mobilization in concert with TCR signaling. ICOS–PI3K-signaling mutant mice produced fewer splenic Tfh cells in response to NP-CGG plus alum immunization, and were unable to promote affinity maturation of NP-specific IgG following a secondary immunization when compared with wild-type mice. Furthermore, it was demonstrated that PI3K activity was required for promoting IL-21 and IL-4 production during *in vitro* T cell stimulation (15).

In addition to the previously characterized roles of catalytic PI3K p110δ subunit recruitment to ICOS, the regulatory p85α subunit of PI3K plays additional roles in driving Tfh cell differentiation. Following ICOS ligation, p85α promotes translocation of osteopontin (OPN) (29), a regulatory protein expressed by activated T cells (93), to the cell nucleus. Within the nucleus, OPN promotes Bcl6 stability by disrupting Bcl6 ubiquitination, thereby reinforcing the Tfh phenotype (29).

Although ICOS-mediated PI3K activity plays an essential role in the process of Tfh cell differentiation, the newly identified ICOS signaling motif IProx has recently been linked to GC Tfh cell commitment. Using TCR Tg SMARTA CD4^+^ T cells expressing a mutant IProx motif within ICOS, Pedros and colleagues demonstrated that these Tg T cells were not defective in early Tfh cell differentiation at day 3 post-LCMV glycoprotein 61-conjugated KLH immunization. However, significant defects in PC, GC B cell, and gp61-KLH-specific isotype-switched Ab production were observed at day 10 post-immunization in mice receiving mutant SMARTA T cells. This IProx motif was further demonstrated to bind the serine–threonine kinase TBK1, and facilitate GC Tfh cell differentiation in concert with conventional ICOS–PI3K signaling. Interestingly, *in vitro* ligation of ICOS alone was insufficient to recruit TBK1 to the IProx motif. Successful ICOS–TBK1 interaction required simultaneous CD3ε and ICOS ligation, suggesting that both TCR and ICOS engagement are necessary for adoption of the GC Tfh phenotype (35). While TBK1 has been shown to be important for induction of type I interferon in innate immune cells (94, 95), its precise role and the additional members of the signaling pathway that promote GC Tfh cell differentiation remain to be defined.

Recently, following a transcriptome analysis designed to identify genes differentially regulated by ICOS, the expression of transcription factor Klf2 was found to be dramatically upregulated after disrupting ICOS signaling. In the absence of ICOS signaling, Klf2 served to decrease CXCR5 expression while increasing CCR7 expression, thereby reversing the Tfh cell phenotype. Ultimately, the decreased expression of Klf2 in Tfh cells was linked to ICOS-mediated repression of Foxo1 (80), a member of the FOXO family of transcription factors responsible for regulating T cell differentiation, as well as numerous other cellular processes (96). In agreement with initiation of the Tfh phenotype via Foxo1 repression, *in vitro* ICOS ligation mediated the nuclear to cytoplasmic translocation of Foxo1. Relief of Foxo1-mediated Bcl6 repression was subsequently shown to consequently promote Tfh cell differentiation. However, Foxo1-deficient CD4^+^ T cells were impaired in their ability to progress to a GC Tfh cell phenotype (81), indicating that ICOS signaling transiently inactivates Foxo1 to allow for initiation of Tfh cell differentiation, but that progression of Tfh cells to a GC Tfh cell phenotype is completed in a Foxo1-dependent manner.

## ICOS and the Germinal Center Reaction

As ICOS co-stimulation plays a critical role in Tfh cell induction and adoption of the GC Tfh cell phenotype, research has also questioned the necessity of ICOS in the production of key GC products, such as MBCs and LLPCs. However, as the absence of ICOS or ICOSL in mice affects multiple steps in the development of T and B cell responses, these mutations cannot be assumed to only influence the GC itself (77). The impact of ICOS on the products of GC reactions, therefore, must be interpreted cautiously. Once thought to be derived solely from the GC reaction, recent evidence indicates that MBC production may not always require GC formation. For instance, early IgM^+^ MBC differentiation in response to TD antigens is largely GC independent (97). While there is evidence that some class-switched MBCs can be produced in the absence of a GC (98), the majority of class-switched MBC formation requires an active GC (99), indicating that ICOS could play an influential role in the production of isotype-switched MBCs. However, while inhibition of ICOS signaling in wild-type mice via α-ICOS Ab administration following NP-CG-alum immunization disrupted early GC formation, it did not impede generation or maintenance of NP-specific IgG1^+^ MBCs between days 10 and 70 post-immunization. However, NIP-binding MBCs from α-ICOS Ab-treated mice accumulated significantly fewer *V_H_186.2* gene mutations relative to control mice between days 30 and 70 post-immunization (98). These studies provided three key insights into MBC production: first, they suggest that isotype-switched MBCs present in α-ICOS-treated mice were mostly likely derived independently of the GC. Second, that over time these early class-switched MBCs are replaced by MBCs derived from a GC reaction that have higher affinity for binding antigen. Finally, despite the lack of somatic hypermutation, the presence of IgG1^+^ NP-specific MBCs in the absence of ICOS signaling suggests that production of class-switched MBCs is not critically reliant on ICOS:ICOSL interactions.

Interestingly, in response to *P. c. chabaudi* AS infection, although *Icos*^−/−^ mice produced fewer IgM^−^ MBCs at week 9 post-infection relative to wild-type mice, class-switched MBC production was not fully abrogated in the absence of ICOS signaling (48). However, the source of these MBCs is still questionable. While the extrafollicular Ab response dominates the humoral response in *P. c. chabaudi* AS-infected *Icos*^−/−^ mice, there is evidence that a small number of GCs are nevertheless formed in the absence of ICOS signaling. Therefore, the isotype-switched MBCs found in *Icos*^−/−^ mice may have a GC origin. Meanwhile, during human systemic lupus erythematosus, ICOS:ICOSL interactions have been linked with aberrant, overproduction of class-switched MBCs (100). Also, human patients with deficiencies in ICOS have significantly reduced numbers of isotype-switched MBCs, but often have normal or slightly reduced numbers of mutated IgM^+^ MBCs (101). Together, these data indicate that early signaling events outside of ICOS:ICOSL interactions are sufficient to promote formation of both IgM^+^ and class-switched MBCs. Nevertheless, without ICOS signaling the number of MBCs generated is reduced, and the production of high-affinity isotype-switched MBCs is impaired, presumably due to disruption of the GC microenvironment.

By facilitating the process of Tfh cell development and promoting the subsequent GC reaction, ICOS:ICOSL signaling may also facilitate production of LLPCs. Interestingly, following DNP-KLH in alum immunization, *Icos*^−/−^ mice were not defective in production of switched DNP-specific IgG or IgG1^+^ splenic and bone marrow-resident LLPCs at week 3 post-immunization when compared with wild-type mice. When re-challenged with DNP-KLH-alum at week 5 post-immunization, however, significantly fewer class-switched antibody-secreting cells (ASCs) were identified in the bone marrow of *Icos*^−/−^ mice at day 5 post-secondary challenge (102), suggesting that secondary PC production is more reliant on ICOS:ICOSL signaling than primary ASC formation.

In the context of *P. c. chabaudi* AS infection, *Icos*^−/−^ mice were not defective in the production of bone marrow-resident, isotype-switched parasite-specific (MSP-1_19_) LLPCs at week 9 p.i., despite a decrease in the quantity and quality of serum MSP-1_19_–IgG relative to wild-type mice at this time point. As *Icos*^−/−^ mice developed dramatically fewer GCs throughout the course of *P. c. chabaudi* AS infection, it is striking that the number of bone marrow-resident ASCs was not significantly impaired (48), as LLPC production is thought to occur relatively late during the GC reaction (99). As an overall decrease in Ab avidity was noted in this study, it is quite possible that a large proportion of the parasite-specific LLPCs seen in the bone marrow of *Icos*^−/−^ mice were derived from the extrafollicular response rather than the GC response, and had homed to the bone marrow relatively early after infection. It is also possible bone marrow-resident ASCs found in *Icos*^−/−^ mice are not long-lived, and are instead continually replaced by splenic plasmablasts as the result of a perpetual extrafollicular response in the absence of GC formation.

Research has also questioned the role of ICOS in the secondary response to previously encountered antigens. Interestingly, disruption of ICOS signaling in wild-type mice via anti-ICOSL Ab during secondary (but not primary) challenge with heat-killed *S. pneumoniae* did not impact boosted PspA-specific IgG Ab titers, suggesting that ICOS is not critical for MBC activation (73). Similarly, following secondary influenza A infection, *Icos*^−/−^ mice were defective in virus-specific IgG2a Ab production but were unimpaired in IgG1 production, harboring only a slight defect in total virus-neutralizing Ab at day 7 post-challenge relative to wild-type mice. Furthermore, splenocytes transferred from wild-type or *Icos*^−/−^ mice previously infected with vesicular stomatitis virus (VSV) induced similar levels of virus-specific IgG in sub-lethally irradiated wild-type recipient mice at day 6 post-VSV infection (103).

However, wild-type mice immunized with phycoerythrin (PE) in alum produced more PE-specific B cells relative to *Icos*^−/−^ mice following secondary PE challenge (102). Similarly, re-challenge of wild-type and *Icosl*^−/−^ mice with NP-CG in alum resulted in higher antigen-specific IgM but lower IgG1 and IgG2a Ab production in *Icosl*^−/−^ mice compared with wild-type mice (66). Although, when NP-primed B cells were isolated from mice treated with or without α-ICOS Ab at the onset of GC formation, no difference in reconstitution of the bone marrow anti-NP IgG1 response was seen upon re-challenge when these B cells were transferred into *Rag-1*^−/−^ mice. However, the ratio of high-affinity NP-specific IgG1 ASCs in the bone marrow was significantly reduced in mice receiving NP-primed B cells from α-ICOS-treated mice (98). These results suggest that ICOS signaling can contribute to the secondary expansion of antigen-specific B cells, particularly by promoting production of isotype-switched high-affinity Abs. The divergent results reported here concerning the necessity of ICOS for MBC activation likely stem from dramatic differences in the source of antigen (i.e., active viral infection versus protein:adjuvant immunization). As alluded to previously, strong inflammatory stimuli, or in this case active viral infection, may overcome a necessity for ICOS signaling in secondary Ab responses.

Although certain aspects of the GC response may be more or less reliant on ICOS signaling depending upon the immunization or infection model in question, these data provide evidence of dysregulated GCs in the absence of ICOS signaling. These defective GC responses may stem from inadequate Tfh cell differentiation, as previously discussed, but could also arise from larger problems in GC architecture. For example, GC B cells isolated from *Icos*^−/−^ mice immunized with NP-CGG exhibit lower expression of lymphotoxin αβ (LTαβ), a molecule upregulated on GC B cells and important in the regulation of the GC microenvironment (104). As LTαβ expression helps maintain a functional FDC network (in part by promoting FDC activation) (105), it is not surprising that impaired LTαβ expression was associated with diminished GC responses (104). Collectively, ICOS:ICOSL signaling promotes critical cellular processes necessary for the production of affinity-matured, class-switched MBCs and LLPCs, but may be less critically important for the initial generation of these cells *per se*.

## ICOS and Memory T Cells

Inducible T cell co-stimulator–ICOSL interactions are important in determining the effector fate decisions of CD4^+^ T cells; however, defective ICOS signaling can also influence the production of memory CD4^+^ T cells. For instance, following priming of mice with Moloney murine leukemia virus envelope protein (H19-env)-pulsed DCs, *Icos*^−/−^ CD4^+^ memory T cells did not expand as abundantly as wild-type memory T cells, despite being retained at similar numbers prior to H19-env immunization (102). Meanwhile, fewer central and effector CD4^+^ memory T cells were observed in *Icosl*^−/−^ or α-ICOS Ab treated wild-type mice 4 weeks after Lm-2W1S infection (106) [attenuated *L. monocytogenes* secreting a chicken OVA-2W1S fusion protein (107)]. Similarly, long-term maintenance of *M. tuberculosis*-specific memory-like CD4^+^ T cells required ICOS expression (108). In a different study, mixed bone marrow chimeras revealed dramatically fewer *Icos*^−/−^ LLOp:I-A^b^-binding T cells (listeriolysin O-specific CD4^+^ T cells) adopted a central memory phenotype at day 20 post-Lm-2W (*L. monocytogenes* producing the 2W peptide and a fragment of listeriolysin O) infection relative to wild-type LLOp:I-A^b^-binding T cells (109). Furthermore, human ICOS deficiency is associated with fewer circulating CD4^+^ memory T cells relative to healthy controls (110). Together, these data suggest ICOS promotes formation and maintenance of a stable population of CD4^+^ memory T cells, and in certain cases may influence their re-activation. However, whether memory CD4^+^ T cell ICOS expression has a direct effect on the ability of MBCs to differentiate into antibody-producing cells or participate in secondary GC reactions during a recall response remains to be determined.

## Conclusion and Implications/Therapeutics

On the whole, ICOS co-stimulation supports a remarkable number of distinct processes during adaptive immune responses. By promoting the induction (15, 29, 71, 78–81), maintenance (29, 48, 79, 80, 87), and follicular homing of Tfh cells (86, 92), ICOS promotes TD Ab responses (37, 38, 65, 66, 71, 72, 76) and drives Ab affinity maturation in the GC reaction (15, 48, 65). Additionally, ICOS serves to enhance or dampen Th1 and Th2 inflammatory responses, depending on the given pathogen. Thus, because impaired ICOS signaling can yield such dramatically different disease outcomes in mice, the role of ICOS in regulating human disease states is of great importance.

In humans, homozygous ICOS deficiency results in common variable immunodeficiency (CVID) (111), a condition characterized by aberrantly low serum gammaglobulin concentration. As such, CVID typically results in frequent bacterial infections of the respiratory and digestive tracts (112). Perhaps not surprisingly, in cases of human ICOS deficiency, there is a dramatic reduction in the frequency of circulating CXCR5^+^ CD4^+^ T cells (113), the presence of which have been linked to active GCs in humans (114–116) and mice (114, 116). Collectively, defects in human ICOS expression correlate with defects in TD Ab production (111), circulating Tfh cells, and GC formation (113), thereby supporting a role for ICOS in human Tfh cell differentiation.

Although human Tfh cells express ICOS (84), a greater frequency of circulating Tfh cells isolated from systemic lupus erythematous (SLE) patients – a disease characterized by autoantibody production (117) – were found to express ICOS relative to healthy controls (116, 118). Interestingly, *in vitro* ICOS co-stimulation of peripheral T cells from patients with active SLE resulted in greatly enhanced IFN-γ production relative to normal controls, and ICOS ligation preferentially induced production of isotype-switched α-double-stranded DNA (α-dsDNA) Abs during co-culture with autologous B cells (118). Similarly, in the murine BXSB-*Yaa* model of SLE, splenic ICOS^+^ CD4^+^ T cell production of IL-21 was linked to renal disease and early mortality (119). In a different study, DC ICOSL expression was correlated with kidney nephritis and proteinuria, as well as kidney-infiltrating T cells, in MRL.*Fas*^lpr^ (lupus-prone) mice (120), further implicating ICOS in promoting tissue inflammation during active SLE. In fact, mutation of roquin – a ubiquitin ligase (121) responsible for post-transcriptionally regulating ICOS mRNA (122, 123) – results in ICOS over-expression on CD4^+^ T cells, thereby promoting spontaneous Tfh cell induction, GC formation, and autoantibody production. Interestingly, unimmunized *sanroque* roquin-mutant mice (*Rc3h1*^san/san^) develop an SLE-like disease, characterized by hypergammaglobulinemia and aberrant IL-21 production (121). As a whole, by way of enhancing autoantibody formation and promoting organ inflammation, these data strongly suggest that ICOS plays a direct role in promoting SLE disease progression in mice and humans.

Not surprisingly, therapeutic interventions designed to disrupt ICOS:ICOSL signaling have been evaluated in mice and humans. The human α-ICOSL antibody AMG 557, for example, proved efficacious in diminishing isotype-switched Ab production, but not IgM production, in SLE patients challenged nasally with KLH in a phase I clinical trial (124). AMG 557 was similarly effective in reducing the production of KLH-specific IgG Abs following intra-dermal immunization with KLH (followed by boost 4 weeks later) in patients diagnosed with mild SLE (125). The results of these studies are promising, and similar success has been achieved in murine model systems. For instance, treatment with α-ICOSL Ab minimized disease symptoms and decreased α-dsDNA Ab production in the NZB/NZW F_1_ model of SLE. Furthermore, in a model of rheumatoid arthritis, α-ICOSL Ab administration suppressed the production of collagen-specific isotype-switched Abs (78). Although these studies indicate neutralization of ICOSL can help ameliorate symptoms associated with SLE, the therapeutic potential of disrupting ICOS signaling may extend beyond TD humoral responses.

Recently, characterization of type 2 innate lymphoid cells (ILC2s), an innate immune cell capable of secreting Th2 type cytokines (126), has revealed that ILC2s express ICOS and ICOSL. Researchers identified a critical role for ICOS:ICOSL signaling in the induction of ILC2-mediated cytokine production that led to airway hyperreactivity (AHR), an indicator of asthma. Interestingly, transfer of human ILC2s into humanized Rag-deficient mice followed by disruption of ICOS signaling via α-ICOSL Ab treatment yielded decreased evidence of IL-33-induced AHR relative to mice receiving isotype control Ab (127). In part, this study suggests novel therapeutic options for AHR could be pioneered with α-ICOS or α-ICOSL Ab treatment.

Along a similar line, α-ICOS Ab therapy has been shown to improve cardiac allograft survival in rats (128–130). However, disruption of ICOS signaling following kidney transplantation in non-human primates was not efficacious in preventing graft rejection (131). Together, these data suggest that ICOS blockade may be more beneficial in combination with traditional drugs (i.e., cyclosporine) used to ameliorate graft-versus-host disease (GVHD). Future studies seeking to gain additional insight regarding the role of ICOS in GVHD could provide efficacious tools to treat chronic graft rejection in humans.

Modulation of ICOS signaling has the potential to mitigate disease severity for a number of human autoimmune disorders. Beyond its key role in isotype-switched Ab production, ICOS can also promote GVHD and potentiate symptoms associated with AHR. Although monoclonal Ab therapy designed to disrupt ICOS:ICOSL interactions appears to be a promising, efficacious treatment, the potential for unintended side effects related to global disruption of TD Ab responses may be unavoidable. Thus, future research concerning how ICOS:ICOSL signals alternatively facilitate Ab production, on the one hand, and T helper cell polarization, on the other, may allow tailoring of treatment options to target a specific disease modality with minimal off-target complications.

## Author Contributions

All authors listed have made substantial, direct, and intellectual contribution to the work and approved it for publication.

## Conflict of Interest Statement

The authors declare that the research was conducted in the absence of any commercial or financial relationships that could be construed as a potential conflict of interest.
